# HMMPolish: a coding region polishing tool for TGS-sequenced RNA viruses

**DOI:** 10.1093/bib/bbad264

**Published:** 2023-07-20

**Authors:** Runzhou Yu, Syed Muhammad Umer Abdullah, Yanni Sun

**Affiliations:** Electrical Engineering, City University of Hong Kong, Tat Chee Avenue, Kowloon, Hong Kong, China; Electrical Engineering, City University of Hong Kong, Tat Chee Avenue, Kowloon, Hong Kong, China; Electrical Engineering, City University of Hong Kong, Tat Chee Avenue, Kowloon, Hong Kong, China

**Keywords:** Third-generation sequencing (TGS), polisher for TGS assemblies, Ribonucleic Acid (RNA) viruses, profile HMMs

## Abstract

Access to accurate viral genomes is important to downstream data analysis. Third-generation sequencing (TGS) has recently become a popular platform for virus sequencing because of its long read length. However, its per-base error rate, which is higher than next-generation sequencing, can lead to genomes with errors. Polishing tools are thus needed to correct errors either before or after sequence assembly. Despite promising results of available polishing tools, there is still room to improve the error correction performance to perform more accurate genome assembly. The errors, particularly those in coding regions, can hamper analysis such as linage identification and variant monitoring. In this work, we developed a novel pipeline, HMMPolish, for correcting (polishing) errors in protein-coding regions of known RNA viruses. This tool can be applied to either raw TGS reads or the assembled sequences of the target virus. By utilizing profile Hidden Markov Models of protein families/domains in known viruses, HMMPolish can correct errors that are ignored by available polishers. We extensively validated HMMPolish on 34 datasets that covered four clinically important viruses, including HIV-1, influenza-A, norovirus, and severe acute respiratory syndrome coronavirus 2. These datasets contain reads with different properties, such as sequencing depth and platforms (PacBio or Nanopore). The benchmark results against popular/representative polishers show that HMMPolish competes favorably on error correction in coding regions of known RNA viruses.

## INTRODUCTION

Ribonucleic acid (RNA) viruses have genetic information encoded in the RNA. These are the most abundant group of parasites that infect at the cellular level [1]. RNA viruses are of huge interest in medical research, as these are the causative agents of a number of diseases such as influenza, hepatitis C, dengue, coronavirus disease 2019 (COVID-19), malaria, etc. Many RNA viruses lack strict proofreading mechanisms and thus can undergo changes or mutations in their genomes during replication. Some mutations enable the virus to evade detection by the host immune system [2–4], and complicate the treatment of the respective disease. Thus, whether it is the study of viral biology or the prediction of mutations related to drug resistance, understanding the viruses at the genome level is of utmost importance.

The genome of an RNA virus is usually smaller than higher organisms and is mostly composed of protein-coding genes. For example, the reference genome, HXB2, of the human immunodeficiency virus (HIV) is $9719$ bases long, out of which $7602$ bases belong to the group-specific antigen (gag), pol, viral infectivity factor (vif), viral protein R (vpr), and env protein-coding genes [5]. The reference genome, H77 (NC004102) [6], of the genotype 1a hepatitis C virus (HCV) is $9646$ bases long, of which the protein-coding genes p22, E1, E2, NS1, NS2, NS3, NS4A, NS4B, NS5A, and NS5B constitute $9012$ bases [7]. These proteins perform specific functions which are critical for the survival and replication of the virus. For example, the protein reverse transcriptase or RNA-dependent deoxyribonucleic acid polymerase is used by some viruses to convert their RNA to deoxyribonucleic acid (DNA) during replication [8]. Inhibition of this protein suppresses viral replication and is used in the treatment of HIV infection [9]. The protein hemagglutinin (HA) enables the influenza virus to bind to cell surface receptors and gain entry into the cell [10]. The gag protein is essential for the assembly of viral particles of HIV [11]. The 3-chymotrypsin-like cysteine protease (3CL$^{\textrm{pro}}$) is required for the cleavage of polyproteins during severe acute respiratory syndrome coronavirus 2 (SARS-CoV-2) replication [12]. Mutations in this protein can result in resistance to the antiviral nirmatrelvir [13]. Detection of specific proteins is used to confirm the presence of viral infection in rapid antigen tests [14, 15]. Understanding viral proteins and their functions is fundamental to rational vaccine design, developing antiviral drugs and suppressing antimicrobial resistance. The low cost and fast processing time of genome sequencing combined with available gene prediction tools make sequencing a popular choice for initial studies of viral proteins. During this process, the accuracy of genome construction or assembly is highly important, as subsequent analysis depends on the quality of the assembled contigs/genomes.

Both next-generation sequencing (NGS) and third-generation sequencing (TGS) are used for viral sequencing. NGS, with Illumina [16] as the main representative, has high throughput and low error rate. The limitations are the read size and the time required for data generation. Compared with NGS, modern TGS platforms can output millions of reads within a few hours. The state-of-the-art TGS technologies are Pacific Biosciences Single Molecule Real-Time (SMRT) sequencing [17] and Oxford Nanopore sequencing [18]. TGS has two main advantages compared with previous technologies. First, TGS is faster as it does not require polymerase chain reaction amplification, which is inherently a time-consuming step. Second, TGS generates reads that are at least an order of magnitude longer than those generated by previous technologies. A TGS platform, MinION, also has the added advantage of portability. MinION has a mass of $90$g [19] and has been used for on-site monitoring of diseases such as Zika [20], Ebola [21], and Yellow Fever [22]. The challenge with the usage of TGS is the accuracy, as the raw per-base error rate is still higher than Illumina. Despite the development of new protocols, the error rates of many existing datasets are still high, particularly in homopolymer regions. RNA sequencing via TGS suffers from even lower accuracy, which has been demonstrated to go as low as ${86\%}$ [23–25].

In this work, we present a pipeline, HMMPolish, to correct (polish) the protein-coding regions of RNA viruses sequenced via TGS. Our tool focuses on known viruses with an established repertoire of reference sequences. The input to the tool consists of the viral contigs obtained via third-party assembly tools or the longest read. The output is the viral sequence with polished protein-coding regions. We demonstrate that among a number of existing polishing tools for TGS data, most fail to correct all errors in the assemblies, some of which lead to incorrect protein translations. Our work addresses this limitation of existing polishers. We first summarize the existing polishing tools for TGS data.

### Related Work

A number of bioinformatics pipelines have been developed to address the accuracy issue of TGS reads [26–33]. These tools use base or $k$-mer frequency as the main feature, and apply statistical or machine learning methods to distinguish errors from true bases. Canu [26] splits the reads into $k$-mers, and constructs a Best Overlap Graph (BOG) [34]. Error correction is performed prior to and during BOG construction. After pre-processing, the BOG is used to generate a corrected assembly, from which contigs are obtained as the maximal non-branching paths. NextPolish [27] computes a confidence score of each base using the frequency of all $3$-mers that include this base. This step is followed by subtracting the sequencing depth at the locus of interest. Once the scores of all bases at all loci have been computed, the base with the maximum score at each locus is kept. Appollo [28] uses an assembler to obtain contigs from the reads. It then uses an aligner to align the reads to the contigs. The contigs obtained in the first step are used to create a profile hidden Markov model (pHMM) graph, and then the alignment obtained in the second step is used to compute the probability of errors in the graph via the Forward–Backward and Baum–Welch algorithms [35]. Finally, the Viterbi algorithm [36] is used to obtain a path in the graph with the minimum probability of error. Raven [30] uses an assembly graph-based approach to correct errors. The idea is that the edges that connect remote parts of an assembly graph likely represent errors or unresolved repeats. Removal of these edges removes the corresponding errors as well. PBDAG-Con [31] selects the longest reads as seeds and aligns all reads to the seeds. Consensus sequences of the aligned reads are generated via a Directed Acyclic Graph (DAG)-based approach in a step referred to as ‘pre-assembly’. The consensus are assembled using an off-the-shelf assembler, and a final consensus is obtained via a maximum likelihood-based approach. Racon [32] aligns reads to a reference and splits the alignment into windows. A Partial Order Alignment graph is constructed from the alignment, from which a consensus sequence is obtained. Medaka [33] computes the base frequency at each locus, and uses these as the input to a multi-layer bidirectional Recurrent Neural Network. The output is the probability of each nucleotide at each locus.

In this work, we deliver a more accurate polishing tool for coding regions of viruses. As viral genomes are generally small and have a high density of protein-coding regions, accurately polished sequences of the protein-coding regions could provide valuable insights about the function of the virus. Our previous work, AccuVIR [37], can polish contigs for both known and novel viruses by the application of *de novo* gene prediction, which may be error-prone for some data. As clinically important viruses have a number of high-quality reference sequences and annotations available, the problem of gene prediction would not pose a challenge when working with such viruses. Here, we build upon our previous work and propose HMMPolish, a polishing tool for the protein-coding regions of known viruses. We focus only on known viruses with multiple reference sequences available to circumvent the issue of *de novo* gene prediction. HMMPolish aligns a read alignment graph and annotated viral protein families. We rigorously tested HMMPolish on a number of real datasets of a number of clinically important viruses. The experimental results show that HMMPolish outperforms state-of-the-art tools in the reduction of errors in protein-coding regions. We also tested HMMPolish on multiple synthetic datasets and the results demonstrate that HMMPolish is robust over a range of data parameters. Thus, we expect that HMMPolish will provide a more accurate picture about virus mutations, which can be an important component in monitoring epidemics.

## METHOD

### Overview of the method

HMMPolish corrects sequencing errors in the coding regions of draft sequences (either raw reads or assembled viral contigs) of known RNA viruses. Insertion and deletion errors (indels) occur frequently in TGS data, especially in homopolymer regions, and can cause frameshifts during translation. Frameshifts, in turn, lead to either incorrect or truncated protein translations. We incorporate protein homology search into our polishing process to rectify the effect of indels and boost the polishing performance.

We formulate the virus coding region polishing as an alignment problem between a pHMM and a sequence alignment graph. In our formulation the pHMM represents a protein family/domain constructed from known variants of a target virus, and the sequence alignment graph is constructed from the reads. The rationale of our approach is that sequencing errors in reads can significantly affect the alignment results between the translated product and the protein family/domain. Thus, if we find a path in the alignment graph with a high pHMM score, this path would be expected to contain fewer errors. We extend the idea of sequence-to-pHMM alignment to graph-to-pHMM alignment. That is, we identify a path in the alignment graph so that its translated sequence can be aligned with the pHMM with maximum score. We obtain the most probable state sequence via the use of an augmented Viterbi algorithm, first proposed in Frame-pro [38]. We also take the path score into consideration in the Viterbi equation to utilize the information contained in the sequence alignment graph. Finally, the sequence represented by the aligned path is output as the polished coding region sequence.

### Profile HMMs for viral protein families

**Figure 1:**
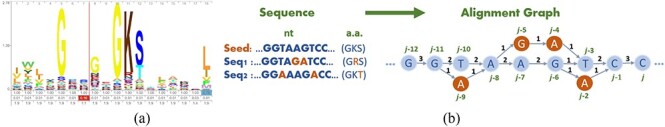
**(a)** Example HMM logo of RNA helicase in norovirus. **(b)** Example of a sequence alignment graph constructed from three sequences. All nodes are numbered in topological order from $j-12$ to $j$.

Profile Hidden Markov Model [39] is one of the most successful models for searching remote homology among protein sequences. A pHMM represents a family/domain of protein sequences. It is a variation of HMM that embeds the position-specific information from a multiple sequence alignment into the states transition diagram [40]. For example, Figure 1(A) shows the HMM logo [41] of the RNA helicase protein in norovirus from Pfam. Columns with high stacks and letters such as G(glycine) and K(lysine) indicate high conservation of these amino acids (a.a.) at corresponding positions.

Pfam, a public protein domain database, contains viral protein families and their corresponding pHMMs for many important viruses such as Influenza A [42], Dengue [43], SARS-CoV-2 [44], Monkeypox [45], etc. Our method takes advantage of the pHMMs of conserved proteins to polish coding regions of RNA viruses.

### Sequence alignment graph

We generate a read alignment graph to store multiple sequences following the steps described in the hierarchical genome-assembly process [31]. The input reads are used to construct a DAG, denoted as $G = (N, E)$, where $N$ and $E$ represent the sets of nodes and edges in the graph, respectively. The nodes in $N$ represent the bases in all reads. If there is a read containing two consecutive bases denoted by nodes $n_i$ and $n_j$, we add a weighted edge $(n_i, n_j)$. The weight represents the number of reads that support the underlying edge.

A toy example is shown in Figure 1(B). After choosing the seed sequence, which could be the longest read or a draft assembly, other reads will be aligned to it for constructing the alignment graph. The selection of the seed sequence influences the graph structure and further influences the Viterbi search result. Thus, we suggest the user select the seed following the criteria: (i) if multiple assemblers generate long contigs, use the longest contig from the assembler with the highest N$50$ value as the seed sequence; (ii) if there are no available contigs from all assemblers, use the longest raw sequence as the seed sequence for alignment graph construction.

### Graph-profile alignment

**Figure 2:**
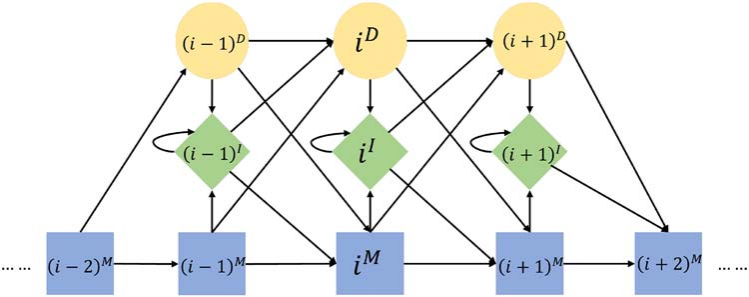
Diagram of a pHMM.

The Viterbi algorithm for sequence-to-pHMM alignment has been elaborated in [46]. We extended the Viterbi algorithm to search a pHMM against a graph [38]. In a standard pHMM, there are three types of states at position $i$, match $i^M$, insert $i^I$ and delete $i^D$ (Figure 2). Only certain transitions among the states are allowed in this HMM: from $i^M$ to $\{i^I,(i+1)^M,(i+1)^D$}, from $i^I$ to $\{i^I,(i+1)^M,(i+1)^D\}$ and from $i^D$ to $\{i^I,(i+1)^M,(i+1)^D\}$. Among all states, only match and insert states will emit a.a. symbols. The start, end and all delete states do not emit any symbols. $e(i^M,x)$ is the emission probability for observing a.a. symbol $x$ at state $i^M$.

$a((j-1)^M, j^M)$ is the transition probability from state $(j-1)^M$ to $j^M$. All the probabilities are in log-space for numerical stability and brevity.

We provide the general Viterbi equation for finding the best alignment for match state $i^M$ below in Eq. 1. The equations for other types of states are available in Supplementary Section 1.1. 


(1) \begin{align*}& V(i^M, j)= e(i^M,x_j) + {\textrm{max}}\left\{ \begin{aligned} V((i-1)^M, j-1) + a((i-1)^M,i^M), \\ V((i-1)^I, j-1) + a((i-1)^I,i^M), \\ V((i-1)^D, j-1) + a((i-1)^D,i^M); \end{aligned} \right. \end{align*}

Nodes in a sequence alignment graph stand for a single base, and are different from the a.a. symbols in protein sequences that are used in sequence-to-pHMM alignment. We thus need to use some extra notations in the graph for graph-profile alignment. In Figure 1(B), we assign topological orders to all nodes based on node $j$ (there might exist more than one topological orders). $S_i$ is the base symbol of node $j$. Every three consecutive nodes $a, b, c$ in the graph form a codon, which we denote by $S_{a}S_{b}S_{c}$. In Figure 1(B), there are two codons ending with node $j$: $S_{j-3}S_{j-1}S_{j}$ ($TCC$) for Serine (S) and $S_{j-2}S_{j-1}S_{j}$ ($ACC$) for Threonine (T).

In sequence-to-pHMM alignment, $V(i^M, j)$ is the maximum log-odds score for aligning the prefix of an input a.a. sequence $\{x_1...x_j\}$ to the pHMM, with a.a. $x_j$ emitted by state $i^M$. For graph-to-pHMM alignment, $V(i^M, j)$ is the maximum score of a path ending with node $j$ against the HMM under the condition that the a.a. translated from a codon with $S_j$ as the third base being emitted by $i^M$. For example, both codons $S_{j-3}S_{j-1}S_{j}$ and $S_{j-2}S_{j-1}S_{j}$ are considered when computing $V(i^M, j)$ in the graph in Figure 1(B). As different paths have different numbers of supporting reads, we also accommodate the path weight in the recursive equation. Let $G(S_{a}S_{b}S_{c})$ be the weight for a codon path $S_{a}S_{b}S_{c}$. With these notations, the optimization goal can be represented as the weighted sum of the Viterbi score and the path score. For better explanation, in Eq. 2 we show the recursive equations for $V(i^M, j)$ using the graph in Figure 1(B) as the reference. It can be easily extended to the general alignment graph. For node $j$, there are two possible prior codons, ‘$TCC$’ formed by $S_{j-3}S_{j-1}S_{j}$ and ‘$ACC$’ formed by $S_{j-2}S_{j-1}S_{j}$. And for codon $S_{j-3}S_{j-1}S_{j}$, transitions from both precedent nodes $j-4$ and $j-6$ should be compared. For brevity, we only show the case for a transition from match state to match state in the Viterbi equation. The complete equation sets are shown in Supplementary Section 1.2. 


(2) \begin{align*}& V(i^M, j)= {\textrm{max}}\left\{\begin{aligned} V((i-1)^M, j-6) + w_1(a((i-1)^M,i^M) + \\ e(i^M, S_{j-2}S_{j-1}S_{j})) +w_{2}(G(S_{j-2}S_{j-1}S_{j})), \\ \\ V((i-1)^M, j-6) + w_1(a((i-1)^M,i^M) + \\ e(i^M, S_{j-3}S_{j-1}S_{j})) +w_{2}(G(S_{j-3}S_{j-1}S_{j})), \\ \\ V((i-1)^M, j-4) + w_1(a((i-1)^M,i^M) + \\ e(i^M, S_{j-3}S_{j-1}S_{j})) +w_{2}(G(S_{j-3}S_{j-1}S_{j})), \\ \\ ......\text{All other paths of three nodes (possible} \\ \text{ codons) ending with }j\text{ in the graph.} \end{aligned} \right. \end{align*}

The weights of the HMM score and path score ($w_1$ and $w_2$) can be adjusted by the user based on the weight values of the constructed graph. In all our experiments, we set $w_1=0.9$ and $w_2=0.1$. These values were chosen to put emphasis on the influence of protein pHMMs. We discussed how the changes of $w_1$ and $w_2$ will affect the results in Supplementary Section 4.1. Users can refer to it when adjusting this parameter based on the input data. Supplementary Section 4.2 provides verbose information in HMMPolish that users can use as a confidence metric to assess the quality of the output and adjust parameters accordingly.

*Running time analysis:* HMMPolish has a time complexity of $O(\beta |N||S|)$, where $\beta $ is the average number of three-base paths ending at each node that need to be considered in the recursive equation. $|N|$ is the number of nodes in $G$, and $|S|$ is the number of states in the pHMM. The primary distinction between HMMPolish and a standard sequence-to-HMM alignment is the introduction of $\beta $, which depends on the coverage of the reads used for graph construction. To reduce the computational cost, graph pruning can be employed as a practical solution, simultaneously reducing both $\beta $ and $|N|$. By removing low-coverage nodes or edges, the graph can be simplified without sacrificing crucial information.

## EXPERIMENTS AND RESULTS

We conducted experiments on both simulated and real TGS datasets. In our experiments on simulated data, we focused on two RNA viruses with high replication rates: influenza-A and HIV-1. In order to evaluate how data properties affect the polishing performance, we simulated TGS data with different sequencing depths, read lengths and sequencing platforms. Then we tested HMMPolish on real TGS data. Although there are many RNA virus sequencing datasets available at the National Center for Biotechnology Information (NCBI) Sequence Read Archive, most datasets lack ground truth information about the underlying genome, making accurate evaluation difficult or unfeasible. We thus validated HMMPolish on datasets with known strain compositions including (i) influenza-A Pacific Biosciences (PacBio) datasets of two subtypes, and six strains for each subtype, (ii) norovirus Nanopore datasets of three samples, (iii) SARS-CoV-2 Nanopore datasets, and (iv) HIV-1 Nanopore datasets. All of our experiments were conducted on a CentOS 7.6 node High Performance Computing Cluster equipped with a 2.7GHz Intel Xeon 6258R CPU and 128 GB memory. Table 1 summarizes the data properties of our experiments.

**Table 1:** Overview of data properties in experiments

			**Simulation parameters**		
	**Virus**	**Sequencing platform**	**Sequencing depth**	**Read length**	**# datasets**
**simulated data**	influenza-A	Nanopore,PacBio	50x,100x,200x	default	6
	HIV-1	Nanopore	50x,100x,200x	2k, 4k, 6k	9
			**Real data attributes**		
	**Virus**	**Sequencing platform**	**Avg. number of reads**	**Avg. read length**	**# datasets**
**real data**	influenza-A	PacBio	6267	1187	12
	norovirus	Nanopore	18 661	1033	3
	SARS-CoV-2	Nanopore	45 629	4050	3
	HIV-1	Nanopore	13 806	1724	1

We benchmarked HMMPolish against popular polishing tools that can be roughly divided into two groups: graph-based tools (Racon [32], PBDAG-Con [31], and AccuVIR [37]) and learning-based tools (Medaka [33]). All these tools require a draft sequence and a set of raw reads as input. Some also require the alignment file between the reads and the draft sequence.

Because HMMPolish focuses on polishing coding regions using pre-built pHMMs of different proteins, our evaluation also focuses on regions covered by the pHMMs only. The evaluation metrics we present include the alignment length of polished sequences, as well as the number of mismatches and indels in polished sequences.

### Experiments on Simulated data

#### Simulated influenza-A data (Nanopore and PacBio)

In our first group of experiments, we tested HMMPolish on simulated influenza-A data with different sequencing depths and from different sequencing platforms. We downloaded the genome of influenza-A subtype H3N2 (strain A/New York/392/2004) from NCBI RefSeq (NC_007366 to NC_007373). Among several long reads simulators such as PBSIM [47], NanoSim [48], SimLoRD [49], and Badread [50], Badread was used in our experiments for data simulation because it is flexible in tuning the attributes such as read length, sequencing depth and error rate. We simulated datasets at different sequencing depths (50x, 100x, and 200x, respectively) and different sequencing platforms (Nanopore and PacBio).

As influenza-A has a segmented genome structure and the shortest segment is below $1000$ bases, the sequenced reads are even shorter than the read length threshold of the long-read assembler. On this group of datasets, none of the long reads assemblers were able to generate a long contig. A naive approach would be to use the longest raw reads from each segment as the seed. However, reads from different segments are mixed, so there was an extra step needed in the seed selection process to distinguish seeds from each segment. We aligned raw reads to the genome of another H3N2 strain (A/Hong Kong/H090-763-V23/2009). Then we selected the reads with the longest alignment on each segment as the seed. Similar steps were applied to the real influenza-A data that we will discuss in later experiments. It is worth mentioning that the specific strain of the reference genome at this step does not influence the results much, as we only need to screen seeds from different segments. For the pHMM model of proteins, we downloaded all available models for influenza-A from Pfam. Supplementary Table S1 shows the pHMMs we used on each segment.

On the influenza-A genome, segment 4 and segment 6 contain the proteins HA and neuraminidase (NA) that characterize different subtypes. We show the performance of different tools on these two proteins and the overall performance on all other six segments. We present the completeness of each output for Nanopore sequencing data in Table 2. HMMPolish was superior to all tools when total uncorrected errors were compared. For reads with $100$x depth, Racon failed to generate any polished output for segments $5$ and $8$. It also shortened the sequence by more than 100 bases in HA. The output of HMMPolish was more complete and accurate. For reads with $200$x depth, Racon had the fewest errors when applied to HA and NA, but suffered from partial completeness. HMMPolish performed the best with four mismatches and eight indels in all other segments.

**Table 2:** Results on simulated H3N2 Nanopore sequencing data for different depths

		**HA**			**NA**			**Other**		**Total**		
	**tool**	**len**	**mis**	**gap**	**len**	**mis**	**gap**	**mis**	**gap**	**mis**	**gap**	Cov_*_
**50x**	**seed**	1822	130	165	1514	67	135	571	838	768	1138	1
	**Medaka**	1773	7	15	1476	4	9	22	119	33	143	1
	**Racon**	1788	27	47	1384	0	6	69	138	96	191	0.98
	**PBDAG-Con**	1758	1	10	1466	0	17	4	110	5	137	1
	**AccuVIR**	1764	2	12	1468	0	11	4	78	6	101	1
	**HMMPolish**	1763	2	12	1465	0	6	12	29	14	47	1
**100x**	**seed**	1825	72	118	1513	106	119	473	819	651	1056	1
	**Medaka**	1767	5	8	1477	6	14	69	171	80	193	1
	**Racon**	1621	0	0	1195	2	6	82	89	84	95	0.79
	**PBDAG-Con**	1757	0	11	1465	1	20	0	74	1	105	1
	**AccuVIR**	1769	0	8	1472	6	6	10	33	16	47	1
	**HMMPolish**	1761	0	7	1465	1	10	3	11	4	28	1
**200x**	**seed**	1788	45	83	1514	71	108	557	958	673	1149	1
	**Medaka**	1768	2	7	1473	0	6	31	101	33	114	1
	**Racon**	1680	0	2	1399	0	0	5	45	5	47	0.97
	**PBDAG-Con**	1762	0	9	1459	1	11	14	98	15	118	1
	**AccuVIR**	1762	0	6	1468	0	9	5	88	5	103	1
	**HMMPolish**	1761	0	2	1459	1	5	4	8	5	15	1

Cov_*_: Coverage of outputs on all segments. This number reflects the completeness of the results by each polisher.

We also present the results on PacBio sequencing data in Supplementary Table S2. By comparing the results in Table 2 and Supplementary Table S2, we can see that although the quality and error pattern of raw reads on the two platforms differ (refer to the seed rows in the tables), there is no obvious advantage or disadvantage for polishers when tested on either platform.

#### Simulated HIV-1 data (Nanopore Sequencing)

HIV-1 has demonstrated high genetic diversity since it was first sequenced in 1960s. In this group of experiments, we simulated a number of Nanopore sequencing datasets from HIV-1 strain 89.6 with different parameters. The reference genome was downloaded from NCBI GenBank (U39362). The simulated sequencing depths were $50$x, $100$x, and $200$x, respectively. The mean lengths were set as $2000$, $4000$ and $6000$ bases, respectively. The genome of HIV-1 has a compact gene structure and high gene diversity with nine genes. Supplementary Table S3 shows the nine pHMMs we used.

**Figure 3:**
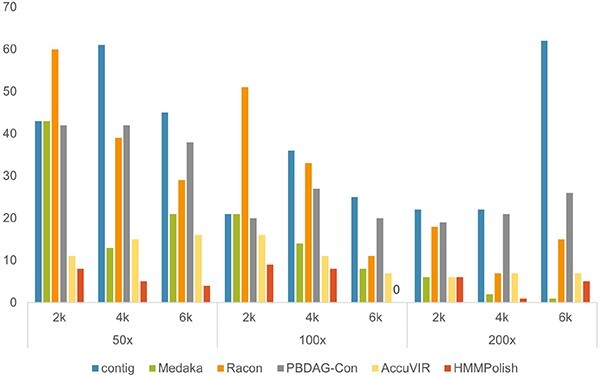
Total errors (sum of errors on four longest genes in Supplementary Table S4) of different tools on simulated HIV-1 datasets.

Because HIV-1 is not a segmented virus, the assemblers are applied and most of them can output a fairly long contig. Therefore, we selected the longest contig of the assembler (Canu, Flye, and wtdbg2) with the highest N$50$ value as the backbone for constructing the read alignment graph. Among the nine genes we polished in the HIV-1 genome, there were four longer than $200$ a.a. (pol, env, gag, and nef). As the errors of all tools on the other five short genes were either zero or one, those did not affect the overall performance comparison. In Figure 3, we show the total errors on the longest four genes of different tools. The results of different tools on each of these four long genes are available in Supplementary Table S4.

HMMPolish ranked the best among all tools on eight out of nine datasets, with errors ranging from $0$ to $9$. AccuVIR ranked No.2 or No.3 for all datasets. When the sequencing depth increased, PBDAG-Con, AccuVIR, and HMMPolish all demonstrated slightly better performance, while Medaka and Racon had remarkable fluctuations. The result of Medaka ranked the second worst with no corrected error on the dataset with lowest depth and shortest read length. Medaka scored the best on the dataset with highest depth and longest read length. Racon might introduce more errors in the polished results on some datasets.

### Experiments on Real data

#### Experiments on 12 real PacBio datasets of influenza-A virus

In this experiment, we selected 12 PacBio datasets from the Influenza Virus Genome Sequencing Project (NCBI SRA accession number: PRJNA183620). Among the 12 datasets, there are two different subtypes, H1N1 and H3N2. Each subtype contains six datasets and every dataset is sequenced from a different strain of that subtype. For subtype H1N1, we selected the seeds for each segment by aligning reads to the genome of the strain A/California/07/2009(H1N1) (NC_026431 to NC_026438). For subtype H3N2, we selected seeds by aligning reads to the genome of the strain A/New York/392/2004(H3N2) (NC_007366 to NC_007373). Table 3 shows the performance of different tools for polishing selected seeds.

**Table 3:** Results on 12 real influenza PacBio datasets.

		**dataset 1**		**dataset 2**		**dataset 3**		**dataset 4**		**dataset 5**		**dataset 6**	
**H1N1**	**tool**	mis	gap	mis	gap	mis	gap	mis	gap	mis	gap	mis	gap
	**seed**	14	190	7	155	11	162	10	301	7	139	14	231
	**Medaka**	0	2	1	5	0	40	0	28	1	1	2	4
	**Racon**	1	7	0	2	6	5	0	1	1	1	1	5
	**PBDAG-Con**	0	0	1	3	3	9	0	2	0	2	0	3
	**AccuVIR**	0	0	1	2	0	0	0	2	0	3	1	5
	**HMMPolish**	0	0	1	0	0	1	0	0	0	0	0	0
		**dataset 7**		**dataset 8**		**dataset 9**		**dataset 10**		**dataset 11**		**dataset 12**	
	**tool**	**mis**	**gap**	**mis**	**gap**	**mis**	**gap**	**mis**	**gap**	**mis**	**gap**	**mis**	**gap**
**H3N2**	**seed**	9	175	13	250	2	105	6	314	2	161	8	292
	**Medaka**	1	7	1	22	0	79	0	20	0	9	0	9
	**Racon**	1	3	1	1	1	2	0	1	0	2	4	5
	**PBDAG-Con**	1	3	8	32	0	3	0	1	0	2	0	1
	**AccuVIR**	1	2	1	0	1	4	0	1	0	2	1	2
	**HMMPolish**	1	2	5	10	0	1	0	1	0	1	0	1

**Figure 4:**
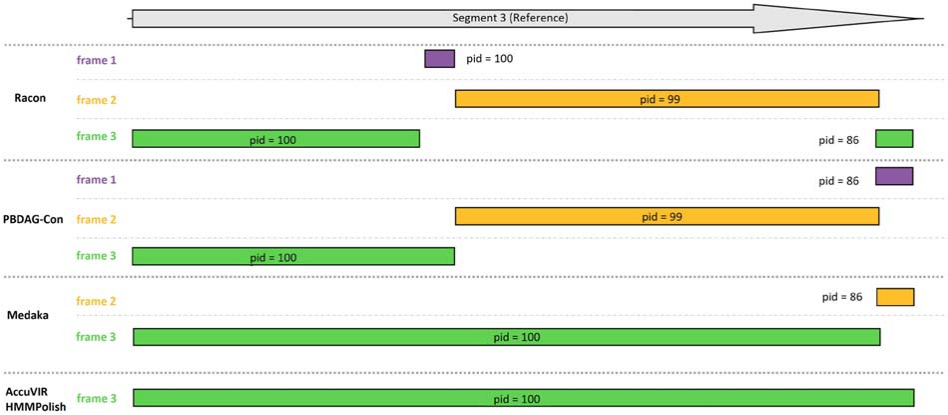
Protein alignment quality of different tools in dataset 6. Racon and PBDAG-Con generated over three partial alignments on three frames. Medaka generates a long and a short alignment on two frames, while AccuVIR and HMMPolish generate a complete protein alignment at the correct translation frame.

In all datasets, the raw seeds contained many errors, especially indels. All tools demonstrated the ability to correct errors in raw sequences, and HMMPolish generated the most accurate results, with fewest errors in the polished outputs. The more detailed results of each dataset are shown in Supplementary Tables S5 and S6. Although the differences between the nucleotide sequences of different tools are slight, there are remarkable changes in the translated sequences. We present this change by showing the alignment of six-frame translated peptide sequences against the real protein product sequence. Figure 4 is an example of the output quality for dataset 6. The influence of gap errors is reflected by the number of frameshifts in the alignment as well as the length and quality of each aligned region.

#### Experiments on real Nanopore sequencing datasets of norovirus

In this experiment, we tested HMMPolish on three real Nanopore sequencing datasets of norovirus. The datasets we used were from BioProject PRJNA713985 [51]. The original project included $39$ samples, and we chose three representative samples (BMH19-145, BMH19-094, BMH19-097) to test different tools. Supplementary Figure S1 shows the sequencing coverage of these three original samples. The first sample (BMH19-145) had high and even sequencing coverage ($\sim 8000$x) along the genome. The second sample (BMH19-094) had uneven coverage on the genome, with $>2000$x depth in the first $2500$ bases and only $\sim 20$x in the rest of the genome. The third sample (BMH19-097) had relatively low but evenly distributed coverage ($>200$x). Besides the differences in coverage distribution, the read lengths also differed among these three samples. Samples $1$ and $2$ both had average read length $<2500$ while sample $3$ had longer average read length ($\sim 4500$). According to the previous experiments, 8000x coverage is an easy case for most tools. Thus, we sampled 10% reads from sample 1 to increase the difficulty level for the following experiment.

For norovirus, seven protein pHMMs were available at Pfam, which covered $>85$% of the genome. Our evaluation also focuses on the quality of regions corresponding to these seven genes. Table 4 displays the comprehensive outcomes obtained using various tools on the three samples. Supplementary Tables S7–S10 provide a detailed analysis of the results obtained for each coding region. For samples $1$ and $2$, assemblers could not generate a complete contig, so we used the longest raw reads as the seeds. For sample $3$, we present the results using both the raw read and the contig as the seed, respectively. On sample $1$, all tools greatly reduced the number of errors in the raw reads as the coverage of the sample was still high enough for polishers ($\sim 800$x after sampled from the original dataset). HMMPolish was able to reduce the error to zero or one on all genes, ranking the best among all tools. On sample $2$, we divided the genes into high and low-depth regions. The high-depth region covered three genes (Shown in Supplementary Section 2.4) and all tools were able to polish the sequence to less than $10$ errors in each gene. In the low-depth region, HMMPolish had the fewest errors for all four genes and was observed to be especially good at reducing gap errors. On sample $3$, the results were similar to sample $1$ when polishing the raw reads, with HMMPolish ranked the best in all coding regions. However, it is worth mentioning that when polishing the high-quality contig, most polishing tools introduced new errors. AccuVIR and HMMPolish were the only two tools that further improved the quality of the polished sequences.

**Table 4:** Results of different tools on three norovirus datasets. Assemblers failed to generate any complete contig for samples 1 and 2, so we used the longest raw reads as the seeds. For sample 3, we present results using both the longest raw read and the contig as seeds.

	**sample 1**			**sample 2**		
**Tool**	**Total mis**	**Total gap**	**Total error**	**Total mis**	**Total gap**	**Total error**
**seed**	323	316	639	304	257	561
**Medaka**	7	24	31	52	55	107
**Raon**	7	7	14	56	34	90
**PBDAG-Con**	2	18	20	39	48	87
**AccuVIR**	0	5	5	51	25	76
**HMMPolish**	2	3	5	48	27	75
	**sample 3 (seed: raw reads)**			**sample 3 (seed: contig)**		
	**Total mis**	**Total gap**	**Total error**	**Total mis**	**Total gap**	**Total error**
**seed**	451	445	896	0	5	5
**Medaka**	6	27	33	6	27	33
**Raon**	0	17	17	0	14	14
**PBDAG-Con**	1	22	23	1	7	8
**AccuVIR**	0	7	7	0	4	4
**HMMPolish**	4	2	6	2	0	2

#### Experiments on real Nanopore sequencing data of SARS-CoV-2

In this experiment, we tested HMMPolish on three real Nanopore sequencing datasets of the spike gene of SARS-CoV-2. The data were sequenced from SARS-CoV-2 patient samples in Hong Kong. Both Illumina sequencing (PRJNA664541) and MinION sequencing (PRJNA664839) were conducted for this project, and we used the *de novo* assembly from Illumina sequencing data as the ground truth for each sample. A detailed description of the assembly process for the Illumina data can be found in Supplementary Section 4.4.

**Figure 5:**
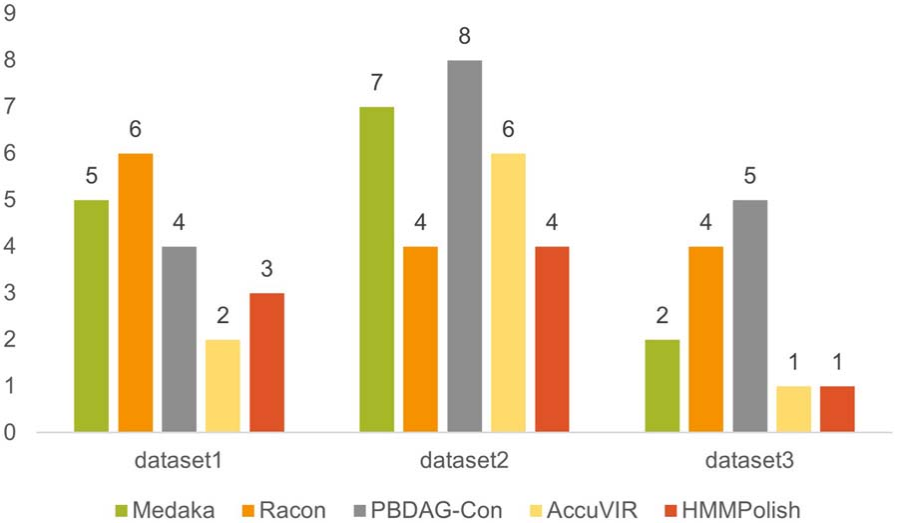
Results of different polishing tools on three real Nanopore datasets of SARS-CoV-2 spike gene.

For SARS-CoV-2 spike gene, there are HMM models of four protein domains: bCoV_S1_N (PF16451.8), bCoV_S1_RBD (PF09408.13), CoV_S1_C (PF19209.3), and CoV_S2(PF01601.19). We show the total errors of different tools in Figure 5. In Supplementary Table S11, we present the quality of different tools when applied to each of the four domains. As domain bCoV_S1_RBD and CoV_S1_C are relatively short ($180$ a.a. and $56$ a.a., respectively), all tools except Racon were able to correct all errors in these two domains. For the domains bCoV_S1_N and CoV_S2, HMMPolish outperformed other polishers on all three datasets, with a maximum error of only one a.a. In Supplementary Table S12, we compared the running time and memory usage of different tools on dataset 1.

#### Experiments on real Nanopore sequencing data of HIV-1

In this experiment, we tested HMMPolish together with other tools on a real Oxford Nanopore Technology (ONT) dataset. The dataset we used was sequenced from HeLa cells infected by HIV-1 strain NL4-3 (GSM4107816). The original project (GSE138425) included samples of different types of cells [52]. We selected this sample because it contained the most HIV-1 reads ($3.9$%) compared with other samples.

In this experiment, the data were from transcriptomes, which means they might have contained all RNA products after the alternative splicing (AS) events in the cellular environment. When aligning these sequences against the reference sequence, splicing sites would result in multiple local alignments if there were remote junctions. As we focus on polishing accuracy, we would neglect the splicing sites by considering the longest local alignments only. We aligned the reads to HIV-1 NL4-3 genome (AF324493.2 in GeneBank) using Minimap2 to extract viral reads for the purpose of assembly and polishing. For the filtered viral sequence set, the average read length was $1700$ bases, and the average depth was above $3000$x. The assembly step generated contigs which are referred to as seed in Table 5. We can see that because of the high coverage of this dataset, the assembled contig had zero mismatch error in the six genes. But gaps existed in eight out of nine genes. After polishing, PBDAG-Con recovered two short proteins (rev and vpr) but had $56$ remaining errors, ranking the worst in all tools. Racon was able to correct a few errors in each protein but only fully recovered vpr. Medaka corrected all errors in four relatively short proteins (nef, rev, tat, and vpr), but left many errors in longer coding regions. HMMPolish outperformed all tools in both the number of fully corrected proteins and the total errors. Only $11$ errors in the gag and pol proteins were not corrected.

**Table 5:** Experiments on real HIV-1 Nanopore datasets (NL4-3)

**protein**	**seed(contig)**			**Medaka**			**Racon**			**PBDAG-Con**			**AccuVIR**			**HMMPolish**		
	**len**	**mis**	**gap**	**len**	**mis**	**gap**	**len**	**mis**	**gap**	**len**	**mis**	**gap**	**len**	**mis**	**gap**	**len**	**mis**	**gap**
**env**	2557	0	9	2557	0	1	2559	3	9	2557	0	6	2557	0	5	2557	0	0
**gag**	1498	0	11	1499	1	4	933	0	4	1498	0	10	1498	0	8	1498	2	0
**nef**	616	0	3	616	0	0	616	0	4	616	0	5	616	0	2	616	0	0
**pol**	3016	10	30	3023	11	16	2611	1	11	3013	9	22	3018	10	22	3007	7	3
**rev**	220	0	1	223	0	0	220	0	1	220	0	0	220	0	1	220	0	0
**tat**	214	4	3	214	0	0	214	1	2	214	0	1	214	0	1	214	0	0
**vif**	574	0	2	575	0	1	574	0	1	574	0	2	574	0	2	574	0	0
**vpr**	286	3	2	286	0	0	286	0	0	286	0	0	286	0	0	286	0	0
**vpu**	226	0	0	226	0	0	226	0	0	226	0	1	226	0	1	226	0	0
**sum**	9207	17	61	9219	12	22	8239	5	32	9204	9	47	9209	10	42	9198	9	3

**Figure 6:**
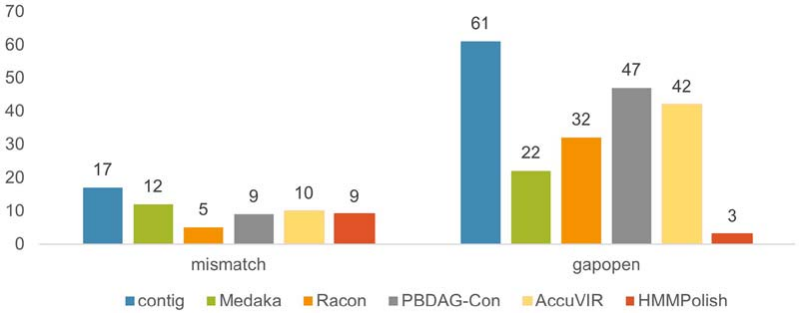
Results of different polish tools on real HIV-1 Nanopore sequencing data.

In Figure 6, we present the numbers of two types of errors (mismatch and gapopen) of different tools in this experiment. HMMPolish shows a clear advantage in reducing the gapopens among other polishers.

## CONCLUSIONS AND DISCUSSIONS

In this study, we introduced HMMPolish, a novel polishing tool that employs base coverage and homology against available viral proteins for further reducing errors in TGS-sequenced RNA viruses. As sequencing errors, particularly insertion/deletion errors, can cause frameshifts in coding regions and thus lead to fragmented or marginal match scores against known proteins, we use alignment against pHMM-encoded protein families to help identify those errors in reads or assemblies. With the reliance on viral protein families, HMMPolish is designed for polishing coding regions of known RNA viruses.

Our approach was evaluated using data from four clinically significant RNA viruses, and we demonstrated that HMMPolish outperformed all state-of-the-art tools in reducing errors in coding regions. The high accuracy provided by HMMPolish is expected to produce more accurate viral strain genomes, which lay the foundation for understanding virus evolution, emergence of new lineages during epidemics, design of vaccine and anti-viral drugs, etc.

In this work, we demonstrated the utility of HMMPolish on segmented viruses. As each segment is polished individually, HMMPolish is compatible to strains with reassortment. If the breakpoints of strains with recombination are located between genes, HMMPolish can be applied to polish the coding regions. It is our future work to extend the utilities of HMMPolish to more general cases for strains with recombinations.

Some RNA viruses with high mutation rates can form quasispecies, which contain multiple haplotypes of extremely high similarity. HMMPolish is not designed to characterize all the haplotypes in a quasispecies. Instead, it will only produce a polished consensus strain. If a user would like to reconstruct all the haplotypes inside quasispecies that are sequenced using TGS, there are specially designed tools for this goal [53, 54]. A recommended approach is to run the haplotype reconstruction tools first. The produced haplotype genomes and their associated reads can be used as input to HMMPolish for polishing.

Another limitation of HMMPolish is that it is not a universal tool and is designed for known viruses with well-curated protein pHMMs. As a result, HMMPolish is not ideally suitable to polish reads for newly discovered viruses unless there are established pHMMs of related proteins.

HMMPolish was developed as a user-friendly tool that is easy to install and use. It has been shown to be applicable to data from different sequencing platforms obtained via different parameters.

**Key Points**

- Accurate reconstruction of viral genomes is important for studies such as mutation inference, understanding virus evolution and lineage classification.
- Despite increased data quality and promising results of error correction tools, many available sequencing data from TGS platforms such as Nanopore still contain errors, especially in homopolymer regions. These errors can lead to over-estimation of viral diversity.
- In this work, we developed HMMPolish, which can polish both assembled viral genomes and raw reads for known viruses. By combining the read coverage information and available protein profile HMMs, HMMPolish achieved the lowest error rate in coding regions across 34 groups of experiments.
- HMMPolish can be applied to produce more accurate viral genomes for clinically important viruses, including HIV-1, influenza-A, norovirus and SARS-CoV-2.

## Supplementary Material

supp_bbad264

## Data Availability

The source code, test data and user manual of HMMPolish are available at https://github.com/rainyrubyzhou/HMMPolish.

## Author Biographies

**Runzhou Yu** is currently pursuing her Ph.D. in the Department of Electrical Engineering at City University of Hong Kong. Her research interest includes sequence analysis in bioinformatics, with a focus on algorithm design for analyzing sequencing data.

**Syed Muhammad Umer Abdullah** is a Ph.D student in the Department of Electrical Engineering at City University of Hong Kong. He has an MPhil in Electronic and Computer Engineering from the Hong Kong University of Science and Technology. His research interests include parameter estimation from evolving populations, algorithm design, and epidemiology.

**Yanni Sun** is an associate professor in the Department of Electrical Engineering at City University of Hong Kong. She got her Ph.D. in Computer Science and Engineering from Washington University in Saint Louis, USA. Her research interests are sequence analysis and metagenomics.
