# Supplementary material for: HMMPolish: a coding region polishing tool for TGS-sequenced RNA viruses
Source: Brief Bioinform. 2023 Jul 20;24(5):bbad264. doi: 10.1093/bib/bbad264 (PMC10516367; doi:10.1093/bib/bbad264)
Supplement: supp_bbad264 [file supp_bbad264.pdf]

HMMPolish: A coding region polishing tool for TGS-sequenced RNA viruses

Runzhou Yu, Syed Muhammad Umer Abdullah, and Yanni Sun

1 Supplementary Equations

1.1 Viterbi equation for finding best sequence-to-pHMM alignment

In the main text, we provide the Viterbi equation for match states only. Below are the equations for insertion and deletion states.

$$V(i^I, j) = e_{i^I}(x_j) + \max \begin{cases} V(i^M, j-1) + a(i^M, i^I), \\ V(i^I, j-1) + a(i^I, i^I), \\ V(i^D, j-1) + a(i^D, i^I); \end{cases}$$

$$V(i^D, j) = \max \begin{cases} V((i-1)^M, j) + a((i-1)^M, i^D), \\ V((i-1)^I, j) + a((i-1)^I, i^D), \\ V((i-1)^D, j) + a((i-1)^D, i^D); \end{cases}$$

1.2 Viterbi equation for finding best graph-to-pHMM alignment

In the main text, we provide the equation representing the transition from a match state to a match state only. Below is the complete Viterbi equation set for the graph-to-pHMM alignment.

$$V(i^M, j) = \max \begin{cases} V((i-1)^M, j-6) + w_1(a((i-1)^M, i^M) + e(i^M, S_{j-2}S_{j-1}S_j)) + w_2(G(S_{j-2}S_{j-1}S_j)), \\ V((i-1)^I, j-6) + w_1(a((i-1)^I, i^M) + e(i^M, S_{j-2}S_{j-1}S_j)) + w_2(G(S_{j-2}S_{j-1}S_j)), \\ V((i-1)^D, j-6) + w_1(a((i-1)^D, i^M) + e(i^M, S_{j-2}S_{j-1}S_j)) + w_2(G(S_{j-2}S_{j-1}S_j)); \\ V((i-1)^M, j-6) + w_1(a((i-1)^M, i^M) + e(i^M, S_{j-3}S_{j-1}S_j)) + w_2(G(S_{j-3}S_{j-1}S_j)), \\ V((i-1)^I, j-6) + w_1(a((i-1)^I, i^M) + e(i^M, S_{j-3}S_{j-1}S_j)) + w_2(G(S_{j-3}S_{j-1}S_j)), \\ V((i-1)^D, j-6) + w_1(a((i-1)^D, i^M) + e(i^M, S_{j-3}S_{j-1}S_j)) + w_2(G(S_{j-3}S_{j-1}S_j)), \\ V((i-1)^M, j-4) + w_1(a((i-1)^M, i^M) + e(i^M, S_{j-3}S_{j-1}S_j)) + w_2(G(S_{j-3}S_{j-1}S_j)), \\ V((i-1)^I, j-4) + w_1(a((i-1)^I, i^M) + e(i^M, S_{j-3}S_{j-1}S_j)) + w_2(G(S_{j-3}S_{j-1}S_j)), \\ V((i-1)^D, j-4) + w_1(a((i-1)^D, i^M) + e(i^M, S_{j-3}S_{j-1}S_j)) + w_2(G(S_{j-3}S_{j-1}S_j)), \\ \text{......All other paths of three nodes (possible codons) ending with j in the graph} \end{cases}$$

$$V(i^I, j) = \max \begin{cases} V((i)^M, j-6) + w_1(a(i^M, i^M) + e(i^M, S_{j-2}S_{j-1}S_j)) + w_2(G(S_{j-2}S_{j-1}S_j)), \\ V((i)^I, j-6) + w_1(a(i^I, i^M) + e(i^M, S_{j-2}S_{j-1}S_j)) + w_2(G(S_{j-2}S_{j-1}S_j)), \\ V((i)^D, j-6) + w_1(a(i^D, i^M) + e(i^M, S_{j-2}S_{j-1}S_j)) + w_2(G(S_{j-2}S_{j-1}S_j)); \\ V((i)^M, j-6) + w_1(a(i^M, i^M) + e(i^M, S_{j-3}S_{j-1}S_j)) + w_2(G(S_{j-3}S_{j-1}S_j)), \\ V((i)^I, j-6) + w_1(a(i^I, i^M) + e(i^M, S_{j-3}S_{j-1}S_j)) + w_2(G(S_{j-3}S_{j-1}S_j)), \\ V((i)^D, j-6) + w_1(a(i^D, i^M) + e(i^M, S_{j-3}S_{j-1}S_j)) + w_2(G(S_{j-3}S_{j-1}S_j)), \\ V((i)^M, j-4) + w_1(a(i^M, i^M) + e(i^M, S_{j-3}S_{j-1}S_j)) + w_2(G(S_{j-3}S_{j-1}S_j)), \\ V((i)^I, j-4) + w_1(a(i^I, i^M) + e(i^M, S_{j-3}S_{j-1}S_j)) + w_2(G(S_{j-3}S_{j-1}S_j)), \\ V((i)^D, j-4) + w_1(a(i^D, i^M) + e(i^M, S_{j-3}S_{j-1}S_j)) + w_2(G(S_{j-3}S_{j-1}S_j)), \\ \text{......All other paths of three nodes (possible codons) ending with j in the graph} \end{cases}$$

$$V(i^D, j) = \max \begin{cases} V((i-1)^M, j) + w_1 a((i-1)^M, i^D)), \\ V((i-1)^I, j) + w_1 a((i-1)^I, i^D)), \\ V((i-1)^D, j) + w_1 a((i-1)^D, i^D)); \end{cases}$$

2 Supplementary Tables

2.1 Experiments on simulated influenza-A Sequencing data

2.1.1 Profile HMMs used for influenza-A

Table S1: Profile HMMs used for influenza-A

segment	proteins	model length	nucleotide length	genome length
seg1	Flu_PB2	754	2262	2316
seg2	Flu_PB1, PB1-F2(2)	819	2457	2317
seg3	Flu_PA	694	2082	2192
seg4	Hemagglutinin	550	1650	1752
seg5	Flu_NP	520	1560	1541
seg6	Neur	334	1002	1432
seg7	Flu_M1_C, Flu_M1, Flu_M2(3)	349	1047	1003
seg8	Flu_NS1, Flu_NS2(2)	311	933	865

2.1.2 Experiments on simulated Influenza-A PacBio Sequencing data

Table S2: Results of simulated H3N2 PacBio sequencing data in different depths.

		HA			NA			Other		Total		
	**tool**	**len**	**mis**	**gap**	**len**	**mis**	**gap**	**mis**	**gap**	**mis**	**gap**	**Cov$_*$**
**50x**	**seed**	1826	75	156	1503	19	60	273	633	367	849	1
	**Medaka**	1795	23	75	1601	7	50	49	141	82	236	1
	**Racon**	1598	0	2	1431	1	4	81	230	10	77	0.95
	**PBDAG-Con**	1745	1	6	1467	0	0	9	71	13	67	1
	**AccuVIR**	1764	2	12	1468	0	0	11	55	10	22	1
	**HMMPolish**	1749	2	5	1466	0	0	8	17	14	47	1
**100x**	**seed**	1761	63	117	1475	31	93	223	560	317	770	1
	**Medaka**	1715	7	12	1482	11	10	78	208	96	230	1
	**Racon**	1595	0	2	1476	1	15	1	29	2	46	0.75
	**PBDAG-Con**	1705	2	5	1467	0	9	60	158	62	172	1
	**AccuVIR**	1702	2	3	1471	3	5	13	54	18	62	1
	**HMMPolish**	1705	2	4	1467	0	4	47	36	49	44	1
**200x**	**seed**	1496	27	84	1367	42	105	234	569	303	758	1
	**Medaka**	1771	4	7	1481	5	10	30	70	39	87	1
	**Racon**	1620	0	2	1253	0	0	2	19	2	21	0.94
	**PBDAG-Con**	1759	2	9	1467	2	8	4	37	8	54	1
	**AccuVIR**	1768	0	5	1470	0	3	8	31	8	39	1
	**HMMPolish**	1758	2	7	1466	1	3	3	8	6	18	1

Cov$_*$: Coverage of outputs on all segments. This number reflects the completeness of the results by each polisher.

2.2 Experiments on simulated HIV-1 Nanopore Sequencing data

2.2.1 Profile HMMs used for HIV-1

Table S3: Profile lengths of HIV-1 genes.

gene	env	gag	nef	pol	rev	tat	vif	vpr	vpu	total
**Amino acid length**	865	493	207	1000	107	101	192	96	82	3143
**Nucleotide length**	2595	1479	621	3000	321	303	576	288	246	9429

2.2.2 Results on simulated HIV-1 Nanopore Sequencing data

Table S4: Results on 4 longest proteins.

Length	tool	2k				4k				6k			
		**env**	**gag**	**nef**	**pol**	**env**	**gag**	**nef**	**pol**	**env**	**gag**	**nef**	**pol**
**50x**	**contig**	14	8	5	16	22	14	7	18	9	13	3	20
	**Medaka**	14	8	5	16	5	3	4	1	3	8	0	10
	**Racon**	24	5	5	26	17	10	4	8	4	10	1	14
	**PBDAG-Con**	15	6	4	16	16	10	5	11	8	12	2	16
	**AccuVIR**	3	2	3	3	5	4	4	3	3	2	1	10
	**HMMPolish**	3	0	1	0	4	1	0	0	1	2	0	1
**100x**	**contig**	2	5	4	10	12	9	3	12	6	6	3	10
	**Medaka**	2	5	4	10	7	6	1	0	2	0	3	3
	**Racon**	8	4	6	33	12	9	4	8	2	3	2	4
	**PBDAG-Con**	3	5	4	8	7	7	2	11	5	5	2	8
	**AccuVIR**	2	5	2	7	5	3	1	2	1	2	2	2
	**HMMPolish**	3	4	0	2	0	3	1	4	0	0	0	0
**200x**	**contig**	3	5	2	12	4	6	3	9	18	10	11	23
	**Medaka**	0	4	0	2	1	0	1	0	0	0	0	1
	**Racon**	5	5	0	8	3	0	2	2	7	0	2	6
	**PBDAG-Con**	2	3	2	12	4	6	2	9	6	6	1	13
	**AccuVIR**	1	1	0	4	0	2	1	4	1	3	0	3
	**HMMPolish**	0	0	0	6	0	0	1	0	1	1	0	3

2.3 Experiments on real influenza-A PacBio datasets

Table S5: Results on six H1N1 PacBio Sequencing datasets

		HA			NA			Other	
	**tool**	**length**	**mis**	**gap**	**length**	**mis**	**gap**	**mis**	**gap**
**dataset 1**	**seed**	1758	1	8	1473	1	42	12	140
	**Medaka**	1752	0	0	1432	0	0	0	2
	**Racon**	1753	1	1	1424	0	0	0	6
	**PBDAG-Con**	1752	0	0	1432	0	0	0	0
	**AccuVIR**	1752	0	0	1432	0	0	0	0
	**HMMPolish**	1752	0	0	1432	0	0	0	0
**dataset 2**	**seed**	1755	0	7	1482	1	49	6	99
	**Medaka**	1752	0	0	1432	0	0	1	5
	**Racon**	1752	0	1	1432	0	0	0	1
	**PBDAG-Con**	1752	0	2	1432	0	0	1	1
	**AccuVIR**	1752	0	1	1432	0	0	1	1
	**HMMPolish**	1752	0	0	1432	0	0	1	0
**dataset 3**	**seed**	1734	7	11	1452	0	22	5	147
	**Medaka**	1729	0	0	1432	0	0	0	40
	**Racon**	1729	0	1	1427	0	0	6	4
	**PBDAG-Con**	1729	0	0	1432	0	0	3	9
	**AccuVIR**	1733	0	0	1432	0	0	0	0
	**HMMPolish**	1729	0	0	1432	0	0	0	0
**dataset 4**	**seed**	1856	7	99	1446	1	17	2	185
	**Medaka**	1762	0	11	1434	0	1	0	16
	**Racon**	1727	0	0	1432	0	0	0	1
	**PBDAG-Con**	1752	0	1	1432	0	0	0	1
	**AccuVIR**	1752	0	1	1432	0	0	0	1
	**HMMPolish**	1752	0	0	1432	0	0	0	0
**dataset 5**	**seed**	1787	0	42	1436	2	5	5	92
	**Medaka**	1752	0	0	1432	0	0	1	1
	**Racon**	1744	0	0	1432	0	0	1	1
	**PBDAG-Con**	1752	0	0	1432	0	0	0	2
	**AccuVIR**	1752	0	0	1432	0	0	0	3
	**HMMPolish**	1752	0	0	1432	0	0	0	0
**dataset 6**	**seed**	1774	1	18	1467	1	36	12	177
	**Medaka**	1752	0	0	1433	0	1	2	3
	**Racon**	1752	0	1	1432	0	1	1	3
	**PBDAG-Con**	1752	0	1	1432	0	0	0	2
	**AccuVIR**	1752	0	1	1432	0	1	1	3
	**HMMPolish**	1752	0	0	1432	0	0	0	0

Table S6: Results on six H3N2 PacBio Sequencing datasets

		HA			NA			Other	
	**tool**	**length**	**mis**	**gap**	**length**	**mis**	**gap**	**mis**	**gap**
**dataset 7**	**seed**	1756	0	16	1453	0	10	9	149
	**Medaka**	1739	0	1	1444	0	0	1	6
	**Racon**	1738	0	0	1444	0	1	1	2
	**PBDAG-Con**	1738	0	0	1444	0	1	1	2
	**AccuVIR**	1738	0	0	1444	0	1	1	1
	**HMMPolish**	1738	0	0	1444	0	1	1	1
**dataset 8**	**seed**	1765	1	30	1450	0	6	12	214
	**Medaka**	1738	0	0	1444	0	0	1	22
	**Racon**	1347	0	0	-	-	-	1	1
	**PBDAG-Con**	1738	0	0	1444	0	0	8	32
	**AccuVIR**	1738	0	0	-	-	-	1	0
	**HMMPolish**	1738	0	0	1444	0	0	5	10
**dataset 9**	**seed**	1749	1	13	1456	0	14	1	78
	**Medaka**	1739	0	1	1445	0	3	0	75
	**Racon**	1738	0	0	1443	0	1	1	1
	**PBDAG-Con**	1738	0	0	1443	0	1	0	2
	**AccuVIR**	1738	0	0	1443	0	1	1	3
	**HMMPolish**	1738	0	0	1443	0	1	0	0
**dataset 10**	**seed**	1749	0	11	1484	1	44	5	259
	**Medaka**	1738	0	0	1445	0	2	0	18
	**Racon**	1738	0	0	1443	0	1	0	0
	**PBDAG-Con**	1738	0	0	1443	0	1	0	0
	**AccuVIR**	1738	0	0	1443	0	1	0	0
	**HMMPolish**	1738	0	0	1443	0	1	0	0
**dataset 11**	**seed**	1784	0	44	1451	1	7	1	110
	**Medaka**	1741	0	3	1443	0	0	0	6
	**Racon**	1738	0	0	1443	0	1	0	1
	**PBDAG-Con**	1738	0	0	1443	0	1	0	1
	**AccuVIR**	1738	0	0	1443	0	1	0	1
	**HMMPolish**	1738	0	0	1443	0	1	0	0
**dataset 12**	**seed**	1773	0	42	1529	1	86	7	164
	**Medaka**	1733	0	1	1445	0	2	0	6
	**Racon**	1732	0	0	1443	0	1	4	4
	**PBDAG-Con**	1732	0	0	1443	0	0	0	1
	**AccuVIR**	1732	0	0	1445	0	2	1	0
	**HMMPolish**	1732	0	0	1443	0	0	0	1

2.4 Experiments on real norovirus Nanopore datasets

Table S7: Results on norovirus dataset 1 (BMH19_145).

	**Calici_PP_N**		**RNA_helicase**		**Peptidase_C37**		**RdRP_1**		**Calici_coat**		**Calici_coat_C**		**RNA_capsid**	
**Tool**	**mis**	**gap**	**mis**	**gap**	**mis**	**gap**	**mis**	**gap**	**mis**	**gap**	**mis**	**gap**	**mis**	**gap**
**seed**	69	59	12	20	103	108	50	44	27	25	16	19	46	41
**Medaka**	1	4	0	2	4	14	1	2	1	1	0	1	0	0
**Raon**	0	0	2	1	4	3	0	2	1	0	0	0	0	1
**PBDAG-Con**	0	6	0	0	0	4	0	5	1	1	0	0	1	2
**AccuVIR**	0	0	0	0	0	4	0	1	0	0	0	0	0	0
**HMMPolish**	0	0	0	0	0	1	0	1	1	0	0	0	1	1

Table S8: Results on norovirus dataset 2 (BMH19_094).

	**Calici_PP_N**		**RNA_helicase**		**Peptidase_C37**		**RdRP_1**		**Calici_coat**		**Calici_coat_C**		**RNA_capsid**	
**Tool**	**mis**	**gap**	**mis**	**gap**	**mis**	**gap**	**mis**	**gap**	**mis**	**gap**	**mis**	**gap**	**mis**	**gap**
**seed**	40	29	6	10	59	54	42	53	84	49	18	14	55	48
**Medaka**	1	5	0	1	6	6	12	9	9	12	10	12	14	10
**Raon**	0	2	0	1	7	4	14	5	12	9	12	8	11	5
**PBDAG-Con**	1	5	0	0	7	1	8	3	5	19	8	6	10	14
**AccuVIR**	0	0	0	0	7	3	9	2	**11**	**6**	11	6	13	8
**HMMPolish**	1	3	0	0	7	2	8	2	8	10	12	5	12	5
							Very low depth region(<20x)							

Table S9: Results on norovirus dataset 3 (BMH19_097 (on raw read)).

	**Calici_PP_N**		**RNA_helicase**		**Peptidase_C37**		**RdRP_1**		**Calici_coat**		**Calici_coat_C**		**RNA_capsid**	
**Tool**	**mis**	**gap**	**mis**	**gap**	**mis**	**gap**	**mis**	**gap**	**mis**	**gap**	**mis**	**gap**	**mis**	**gap**
**seed**	66	78	8	21	117	112	93	88	57	51	38	39	72	56
**Medaka**	0	5	0	1	0	7	2	4	0	2	2	4	2	4
**Raon**	0	2	0	1	0	6	0	1	0	2	0	3	0	2
**PBDAG-Con**	0	5	0	0	0	3	0	2	1	2	0	5	0	5
**AccuVIR**	0	2	0	0	0	1	0	0	0	2	0	1	0	1
**HMMPolish**	1	1	0	0	1	0	0	0	1	0	1	0	0	1

Table S10: Results on norovirus dataset 3 (BMH19_097 (on contig)).

	**Calici_PP_N**		**RNA_helicase**		**Peptidase_C37**		**RdRP_1**		**Calici_coat**		**Calici_coat_C**		**RNA_capsid**	
**Tool**	**mis**	**gap**	**mis**	**gap**	**mis**	**gap**	**mis**	**gap**	**mis**	**gap**	**mis**	**gap**	**mis**	**gap**
**seed**	0	0	0	0	0	1	0	0	0	1	0	2	0	1
**Medaka**	0	5	0	1	0	7	2	4	0	2	2	4	2	4
**Raon**	0	1	0	1	0	3	0	1	0	2	0	4	0	2
**PBDAG-Con**	0	1	0	0	0	1	0	0	1	1	0	2	0	2
**AccuVIR**	0	0	0	0	0	1	0	0	0	1	0	1	0	1
**HMMPolish**	1	0	0	0	0	0	0	0	1	0	0	0	0	0

2.5 Results on three SARS-CoV-2 spike gene datasets

Table S11: Results on three SARS-CoV-2 spike datatsets.

len	tool	bCoV_S1_N		bCoV_S1_RBD		CoV_S1_C		CoV_S2		total	
		mis	gap	mis	gap	mis	gap	mis	gap	mis	gap
**dataset 1**	seed	14	18	4	8	4	2	22	24	44	52
	Medaka	1	0	0	0	0	0	1	3	2	3
	Racon	1	0	0	1	0	4	0	0	1	5
	PBDAG-Con	1	2	0	0	0	0	0	1	1	3
	AccuVIR	1	1	0	0	0	0	0	0	1	1
	HMMPolish	1	2	0	0	0	0	0	0	1	2
**dataset 2**	seed	23	30	18	20	1	4	37	65	79	119
	Medaka	1	1	0	0	0	0	1	4	2	5
	Racon	0	0	0	0	0	0	0	4	0	4
	PBDAG-Con	0	3	0	0	0	0	0	5	0	8
	AccuVIR	0	2	0	1	0	0	0	3	0	6
	HMMPolish	0	1	0	0	0	0	0	3	0	4
**dataset 3**	seed	4	8	7	10	0	6	25	44	36	68
	Medaka	1	0	0	0	0	0	0	1	1	1
	Racon	1	1	0	0	0	0	0	2	1	3
	PBDAG-Con	1	2	0	0	0	0	0	2	1	4
	AccuVIR	1	0	0	0	0	0	0	0	1	0
	HMMPolish	1	0	0	0	0	0	0	0	1	0

Table S12: Running time and memory usage comparison (on SARS-CoV-2 spike dataset 1).

**Tools**	**Running Time**(hh:mm:ss)	**Memory Usage**(MB)
Medaka	00:01:43	355
Racon	00:14:29	371
PBDAG-Con	00:01:33	1,928
AccuVIR	00:07:14	3,108
HMMPolish	00:04:44	4,632

The comparison was conducted using real Nanopore Sequencing data of SARS-CoV-2, with a dataset of 191MB containing 48,905 reads. Among the five tools evaluated, HMMPolish ranked third in running time, outperformed by the learning-based tool Medaka and the consensus-based tool PBDAG-Con. The memory usage of the three alignment graph-based tools was higher than that of the other two tools. However, this can be optimized and mitigated by pruning low-weight edges in the graph. We will continue to optimize the software in our near future work.

3 Supplementary Figures

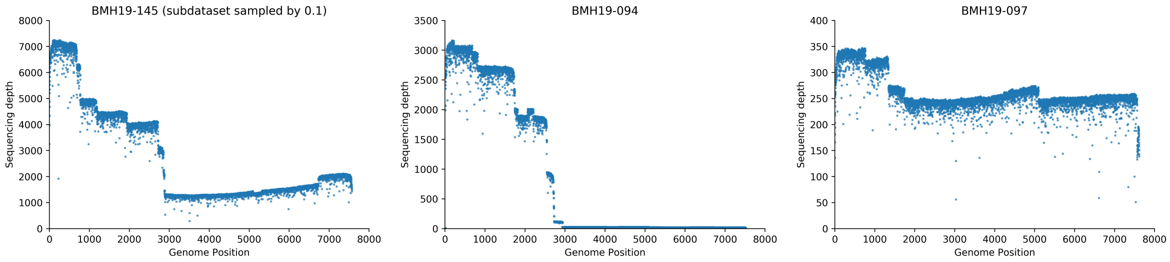

Figure S1: Sequencing depths of the original three norovirus datasets.

4 Supplementary Information for Experiments

4.1 Influence of the weights of the HMM score and path score

An important factor to consider is the quality of the pHMM for graph searching, as using a low-quality pHMM can result in a high number of errors. For example, the pHMM may be trained using lowly-conserved sequences or a high taxonomy diversity, rather than being designed for the targeted viral protein. Another scenario is when the pHMM is highly conserved, but the training protein cluster shows low similarity with the target viral genes.

To illustrate this issue, we present the results of using models from Pfam (accession number: PF00075) to polish simulated HIV-1 data in Figure. S2. This model represents the Ribonuclease H protein family but has diverse taxonomy compositions, with only around 1/3 of the training sequences coming from viruses. Under this setting, using HMM scores alone ($w_2 = 0$) results in sequences containing over 40 errors. However, when we combine the path score with the optimization function, the number of errors significantly decreases. Our preliminary experiments show that the best setting is $w_1 = 0.9$ and $w_2 = 0.1$, which is also the default weight value set for HMMPolish.

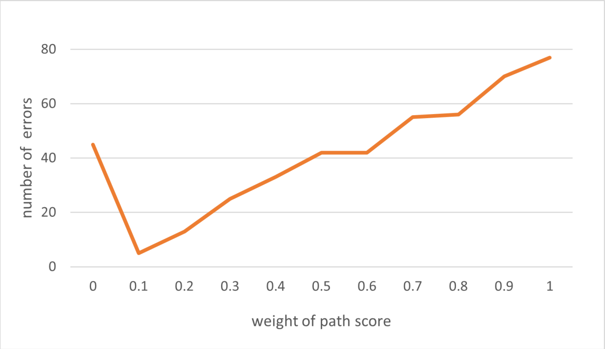

Figure S2: Impact of changing the weight of path score (w_2) on the quality of the output in HIV-1 data.

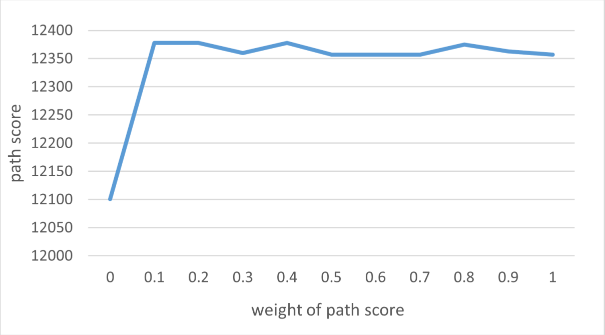

Figure S3: Impact of changing the weight of path score (w_2) on the quality of the output in norovirus dataset 2

Another important factor to consider is the coverage of reads, as the path score value is highly dependent on the edge weights, which quantify the reads coverage. In the recursive score equation, we deduct the backbone edge score when calculating the three-base path score to normalize it with the HMM score. However, when the reads coverage is exceptionally high, the path score may become dominant, and adjusting the w_1 and w_2 weights may not make any significant difference in the results.

To illustrate this issue, we present the results of the real Nanopore sequencing data of norovirus (dataset 2 in the experiment). Figure. S3 shows that, except for the obvious increase in the path score when w_2 increases from 0 to 0.1, the path score only has minor fluctuations with the increase of w_2.

4.2 Metrics for the quality of HMMPolish output

HMMPolish has a "verbose" mode that provides additional information to assess the quality of the polished coding region. The two metrics provided are:

1. The ratio between the polished path weight and the consensus path weight: This metric provides a measure of confidence in the quality of the polished region. A higher ratio indicates a higher level of confidence. The path weights are quantified using the consensus path generated by DAG-Con.

2. The bitscore: This metric is obtained by aligning the pHMM to the polished sequence using HMMER (1) and provides a measure of the similarity between the polished sequence and the pHMM. Users can compare this score with the bitscore of a genome sequence on the same pHMM to evaluate the quality of the polished sequence.

These additional metrics are useful as they provide an objective way to evaluate the quality of the polished sequences, which enhances the utility of HMMPolish. Users can use the confidence scores to assess the quality of the polished coding region and make informed decisions about the downstream analysis.

4.3 Assembly of SARS-CoV-2 data from Illumina reads

The Illumina reads were in FASTQ format and were aligned to the ancestral sequence of SARS-CoV-2 (NCBI accession: NC_045512) using the "index" and "mem" commands of BWA (2).

The resulting aligned files were then sorted and converted to binary format, producing sorted Binary Alignment Map (BAM) files.

Finally, the consensus sequence for each dataset was obtained using a combination of tools, including SAMtools mpileup, BCFtools, vcfutils.pl (3), and SEQTK (4). Detailed command files for the entire process can be found on our GitHub page (`https://github.com/rainyrubyzhou/HMMPolish`).

4.4 Versions for related tools

- Canu: V2.1.1
- Medaka: V1.6.0
- Racon: V1.3.0
- PBDAG-Con: V0.2.3
- AccuVIR: V1.0
- BWA: V0.7.17
- SAMtools: V1.8
- BCFtools: V1.17
- SEQTK: V1.3

4.5 Command Parameters

Medaka for running on reads.fa:

medaka_consensus -i reads.fa -d draft_seq.fa -o medaka_out

Racon for running on reads.fa:

minimap2 -x ava-ont draft.fa reads.fa > align.paf
racon reads.fa align.paf draft.fa > racon_out.fa

PBDAG-Con for running on reads.fa:

blasr reads.fa backbone.fa -bestn 1 -m 5 -out mapped.m5
pbdagcon mapped.m5 > output.fa

AccuVIR for running on reads.fa:

python AccuVIR_main.py -r reads.fa -b backbone.fa
python AccuVIR_MRR.py -r paths.fa

HMMPolish for running on reads.fa:

python HMMPolish.py –read reads.fa –seed seed.fa –hmm profiles.hmm

Illumina commands

Align FASTQ files to reference:

bwa index NC_045512.fasta
bwa mem NC_045512.fasta <FILE>.fastq.gz > FILE.sam

Sort aligned SAM files:

samtools faidx NC_045512.fasta
samtools view -bt NC_045512.fai <FILE>.sam > <FILE>.bam
samtools sort <FILE>.bam > <FILE>_sorted.bam

Deduplicate sorted BAM files:

java -jar picard.jar MarkDuplicates REMOVE_DUPLICATES=true I=<FILE>_sorted.bam O=<FILE>.bam M=<FILE>_metrics.txt

Generate consensus sequence in FASTQ format:

samtools mpileup -uf NC_045512.fasta <FILE>.bam | bcftools call -c | vcfutils.pl vcf2fq > consensus_fq.fastq

Generate consensus sequence in FASTA format:

seqtk seq -aQ64 -q20 -n N consensus_fq.fastq > consensus.fasta

References

[1] R. D. Finn, J. Clements, and S. R. Eddy, "Hmmer web server: interactive sequence similarity searching," Nucleic acids research, vol. 39, no. suppl_2, pp. W29–W37, 2011.

[2] H. Li, "Aligning sequence reads, clone sequences and assembly contigs with bwa-mem," 2013.

[3] P. Danecek, J. K. Bonfield, J. Liddle, J. Marshall, V. Ohan, M. O. Pollard, A. Whitwham, T. Keane, S. A. McCarthy, R. M. Davies, and H. Li, "Twelve years of SAMtools and BCFtools," GigaScience, vol. 10, 02 2021. giab008.

[4] H. Li, "seqtk, toolkit for processing sequences in fasta/q formats." `https://github.com/lh3/seqtk`, 2012.
